# Disease activity of lung cancer at the time of acute exacerbation of interstitial lung disease during cytotoxic chemotherapy

**DOI:** 10.1111/1759-7714.14566

**Published:** 2022-07-15

**Authors:** Akimasa Sekine, Goushi Matama, Eri Hagiwara, Erina Tabata, Satoshi Ikeda, Tsuneyuki Oda, Ryo Okuda, Hideya Kitamura, Tomohisa Baba, Hiroaki Satoh, Toshihiro Misumi, Shigeru Komatsu, Tae Iwasawa, Takashi Ogura

**Affiliations:** ^1^ Department of Respiratory Medicine Kanagawa Cardiovascular and Respiratory Center Yokohama Kanagawa Japan; ^2^ Department of Internal Medicine, Mito Medical Center University of Tsukuba Mito Japan; ^3^ Department of Biostatistics Yokohama City University School of Medicine Yokohama Japan; ^4^ Department of Radiology Kanagawa Cardiovascular and Respiratory Center Yokohama Kanagawa Japan

**Keywords:** acute exacerbation, disease activity, interstitial lung disease, lung cancer

## Abstract

**Background:**

The prognosis of lung cancer patients with interstitial lung disease (ILD) is poor, and acute exacerbation (AE) of ILD can occur during chemotherapy as a fatal adverse event. Although AE‐ILD development is correlated with various factors, no reports are investigating the disease activity of lung cancer at the time of AE‐ILD development.

**Methods:**

All consecutive lung cancer patients with ILD who developed chemotherapy‐related AE‐ILD within 28 days after the last administration of cytotoxic chemotherapy between 2011 and 2020 were retrospectively reviewed.

**Results:**

Among 206 lung cancer patients with ILD who were treated with cytotoxic chemotherapy, 30 patients were included. The median age was 72 years and all patients were men with smoking history. Usual interstitial pneumonia (UIP) and non‐UIP patterns of ILD was observed in 17 and 13 patients. Most of AE‐ILD occurred during second‐ or later‐line (22/30, 73.3%) and developed within first or second courses during chemotherapy (19/30, 63.3%). Regarding tumor response to chemotherapy at AE‐ILD development, majority of patients (18 patients, 60.0%) experienced progressive disease and only one patient (3.3%) experienced a partial response. Notably, 27 patients (90.0%) did not exhibit any tumor shrinkage of the thoracic lesions.

**Conclusion:**

Lung cancer was uncontrolled with cytotoxic chemotherapy at the time of AE‐ILD development. Although AE‐ILD during chemotherapy has been generally discussed in terms of drug‐specific adverse effects, uncontrolled lung cancer may be also correlated with AE‐ILD development.

## Graphical abstract

This study retrospectively included 30 consecutive lung cancer patients who developed acuate exacerbation (AE) of interstitial lung disease (ILD) during cytotoxic chemotherapy, and investigated the disease activity of lung cancer at the time of AE‐ILD development. Regarding change in tumor size of thoracic lesion from baseline or best response, majority of patients (18/30, 60%) experienced progressive disease. Only a patient (3.3%) presented a partial response. Notably, 27 patients (90%) did not exhibit any tumor shrinkage of the thoracic lesions. Although AE‐ILD during cytotoxic chemotherapy has been generally discussed in terms of drug‐specific adverse effects, uncontrolled lung cancer may be also correlated with AE‐ILD development.

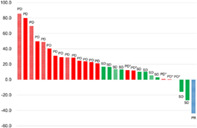

## INTRODUCTION

Interstitial lung disease (ILD) is characterized by various degrees of inflammation and fibrosis,^1^ and the presence of ILD is widely accepted as a risk factor for lung cancer development.^2^, ^3^, ^4^ Lung cancer patients with ILD reportedly present with a very poor prognosis and acute exacerbation (AE) of ILD can sometimes develop during chemotherapy as a fatal adverse event.^5^, ^6^, ^7^, ^8^ As a result, multiple studies have investigated the safety and efficacy of therapeutic regimens for lung cancer patients with ILD.^9^, ^10^, ^11^, ^12^, ^13^, ^14^, ^15^, ^16^ Although AE‐ILD development is reported to be correlated with various factors, including low vital capacity, radiologically usual interstitial pneumonia (UIP) pattern, and non‐small cell lung cancer (NSCLC),^17^, ^18^, ^19^ no reports have investigated the disease activity of lung cancer at the time of AE‐ILD development. We speculated that the tumor response itself is inversely correlated with AE‐ILD development. Therefore, this retrospective study verifies the above‐mentioned hypothesis.

## METHODS

### Patients

We retrospectively investigated all lung cancer patients with ILD who were treated with systemic cytotoxic chemotherapy between January 2011 and December 2020. ILD was diagnosed based on medical history, physical examination, and radiological abnormalities compatible with bilateral lung fibrosis including ground‐glass opacity, consolidation, and/or reticular shadow. Patients who developed cytotoxic chemotherapy‐related AE‐ILD were included. As per the methods in previous studies, this study included UIP pattern ILD and non‐UIP pattern ILD. UIP or probable UIP was defined as UIP pattern ILD, while the other type of ILD was defined as non‐UIP pattern ILD based on the International Consensus Statement.^20^ The radiological diagnosis of ILD and AE‐ILD was made based on the consensus of at least two board‐certified chest physicians (A.S., T.B., O.T., R.O.) and chest radiologist (T.E.). Patient characteristics including sex, age, smoking history, performance status, tumor histology, disease stage, radiological classification, treatment history and results of laboratory data including C‐reactive protein (CRP), Krebs von den Lungen‐6 (KL‐6) and surfactant protein‐D (SP‐D) at the time of initiation of last course of chemotherapy were investigated.

### Chemotherapy‐related AE‐ ILD and evaluation of disease activity

Chemotherapy‐related AE‐ILD was confirmed if all of the following four criteria were met according to previous reports^1^, ^17^, ^21^: acute worsening or development of dyspnea^2^; high‐resolution computed tomography (HRCT) findings indicating new bilateral ground‐glass attenuations with/without nonsegmental consolidation superimposed on pre‐existing interstitial shadows^3^; deterioration not fully explained by cardiac failure or fluid overload, on the basis of the results of biochemical tests and echocardiography and subsequent clinical course; and^4^ <4 weeks interval between the last administration of chemotherapeutic drugs and the onset of AE‐ILD. In addition, patients who developed AE‐ILD immediately after or during radiotherapy or immune‐checkpoint inhibitors were excluded because radiation pneumonitis and pseudoprogression could not be completely denied.^22^ At the diagnosis of AE‐ILD, the percent change in intrathoracic lesion size from baseline or best response was evaluated using Response Evaluation Criteria in Solid Tumor (RECIST) version 1.1.^23^

### Overall survival

Overall survival (OS) after AE‐ILD diagnosis and after initiating first line chemotherapy was investigated in all patients who developed chemotherapy‐related AE‐ILD. In addition, OS was also evaluated in all patients who did not develop any AE‐ILD during their clinical courses. Death of chemotherapy‐related AE‐ILD was defined as death within 28 days of AE‐ILD development because a previous report showed that about 60% of patients with idiopathic pulmonary fibrosis (IPF) died within a month of AE development.^24^

### Statistical analysis

Descriptive statistics were expressed as *n* (%) or median and range. Fisher's exact test or chi‐squire test was used to compare categorical variables and the Mann–Whitney U test was performed to compare continuous variables. Overall survival was defined as the period from the diagnosis of chemotherapy‐related AE‐ILD or the initiation of first‐line chemotherapy to the day of death of any cause, using the Kaplan–Meier method. The outcome was censored if a patient had not died at the time of the last follow‐up. JMP 10 software (SAS Institute) was used for all statistical analysis. This study was approved by the Institutional Ethical Review Board (IRB) of Kanagawa Cardiovascular Respiratory Center, Yokohama, Japan (IRB: KCRC‐19‐0020).

## RESULTS

### Patient characteristics at the time of last chemotherapy immediately before AE‐ILD

**FIGURE 1:**
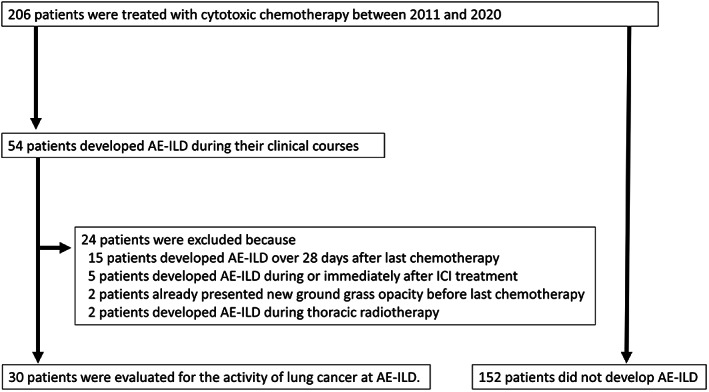
Study flow.

Figure 1 shows the study flow. A total of 206 patients with ILD were treated with cytotoxic chemotherapy. During their clinical courses, 54 patients developed AE‐ILD and 152 patients did not. Among 54 patients who developed AE‐ILD, 30 patients were confirmed as developing cytotoxic chemotherapy‐related AE‐ILD. Table 1 shows the patient characteristics at the time of initiating last course of chemotherapy. All patients were male and smokers with a median age of 72 years. In most patients (25/30), performance status was 0―1. The UIP pattern of ILD was observed in 20 patients (66.6%), whereas non‐UIP was present in 10 patients (33.3%). The histological subtypes of the tumors were adenocarcinoma in 10 patients, squamous cell carcinoma in seven patients, neuroendocrine cell cancer in seven patients, and other nonspecified tumors in six patients. The disease stage was stage 3 in 13 patients, stage 4 in 14 patients, and three patients experienced postoperative recurrence. With regards to cause of ILD, 29 patients had idiopathic ILD. More than half of patients had comorbidity such as diabetes mellitus and hypertension. The median level of KL‐6, SP‐D and CRP were 938 U/ml, 177.1 ng/ml and 2.85 mg/dl, respectively. Regarding previous treatment, three patients received immune‐checkpoint inhibitors and one patient underwent thoracic radiotherapy.

**TABLE 1:** Patient characteristics at the initiation of last course of chemotherapy immediately before AE‐ILD

	All patients (*n* = 30)	NSCLC (*n* = 23)	NEC (*n* = 7)
Sex (male/female)	30/0	23/0	7/0
Age (range [years])	72 (55–80)	72 (58–80)	70 (55–79)
PS (0–1/>2)	25/5	20/3	5/2
Smoking status (current/former)	12/18	10/13	2/5
Pack‐year	45.5 (20–100)	48 (20–100)	45 (25–75)
Radiological classification (UIP/non‐UIP)	20/10	15/8	5/2
Pathology (Ad/Sq/NOS/NEC)	10/7/6/7	10/7/6/0	0/0/0/7
Stage (III/IV/recurrence^a^)	13/14/3	10/11/2	3/3/1
Cause of ILD			
Idiopathic/CTD	29/1	22/1	7/0
Comorbidity			
Diabetes mellitus	7	7	0
Hypertension	7	5	2
CTD	1	1	0
Hepatic disease	2	2	0
Angina pectoris	2	2	0
None	11	7	5
Laboratory test			
KL‐6 (*n* = 29)	938 (418–4991)	957 (437–3263)	816 (418–4991)
SP‐D (*n* = 16)	177.1 (60.5–283.9)	173.8 (60.5–293.9)	211.1 (142.8–234.5)
CRP (*n* = 30)	2.85 (0.27–26.5)	3.37 (0.27–26.5)	1.85 (0.6–2.56)
Treatment history			
Immunocheckpoint inhibitor (yes/no)	3/27	3/20	0/7
Thoracic radiotherapy (yes/no)	1/29	1/22	0/7

Abbreviations: AE‐ILD, acute exacerbation‐interstitial lung disease; CRP, C reactive protein; CTD, connective tissue disease; KL‐6, Krebs von den Lungen‐6; NEC, neuroendocrine cell carcinoma; NOS, not other‐specified; NSCLC, non‐small cell lung cancer; PS, performance status; SP‐D, surfactant protein‐D; UIP, usual interstitial pneumonia.

^a^ Postoperative.

### Treatment regimen and disease activity of lung cancer at the time of AE diagnosis

**FIGURE 2:**
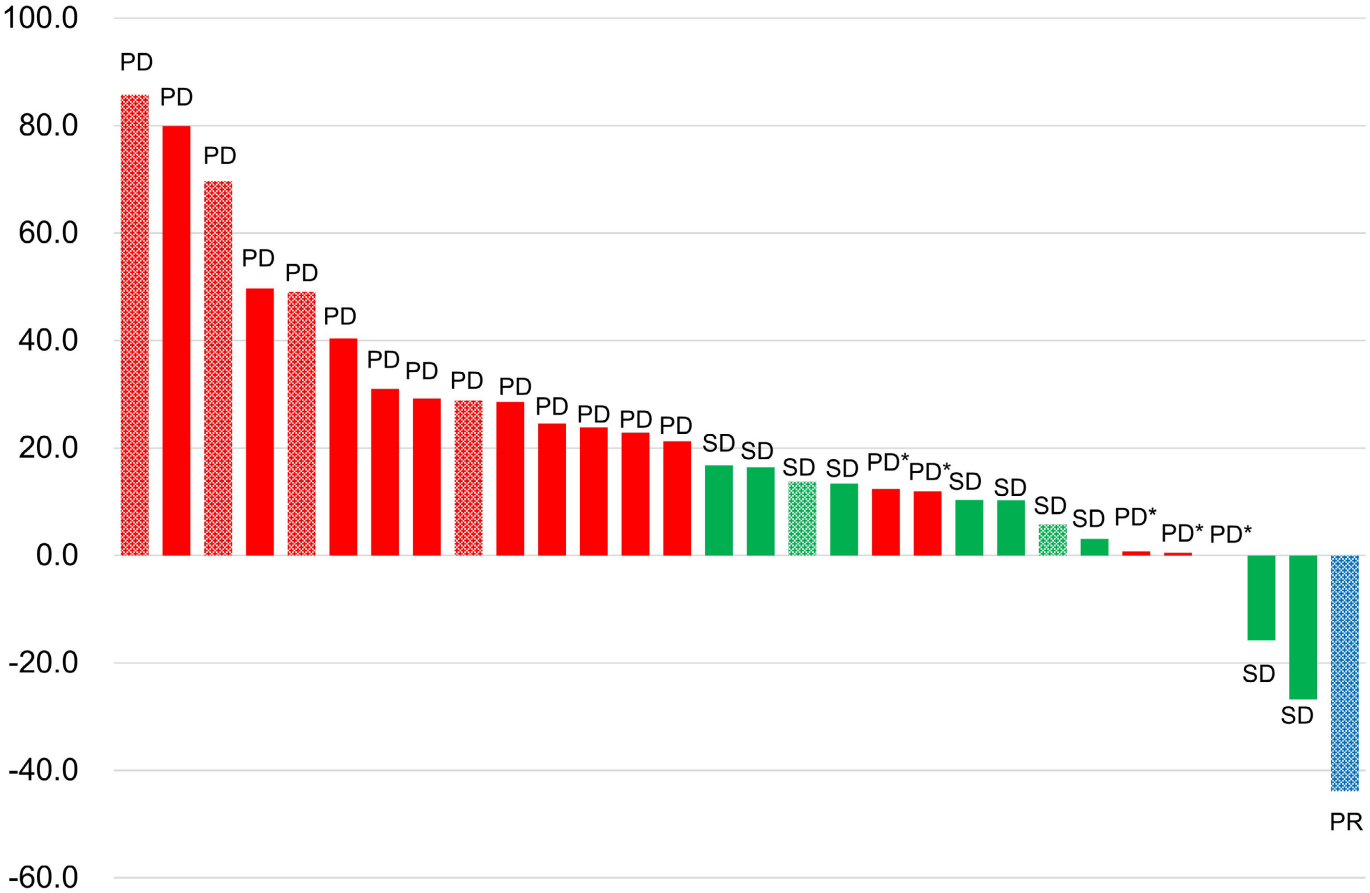
The waterfall plot for tumor response and percent change in the intrathoracic lesion size from baseline or best response at the time of acute exacerbation of interstitial lung disease. PD, progressive disease; PR, partial response; SD, stable disease; *PD confirmed due to distant metastases and/or malignant pleural effusion. Shading indicates neuroendocrine cell carcinoma.

AE‐ILD occurred during first‐line treatment in eight patients, second‐line treatment in 11 patients, third‐line treatment in seven patients and fourth‐line treatment or later in four patients, as shown in Table 2. Thus, AE‐ILD mostly developed during second‐ or later‐line chemotherapy (22/30, 73.3%). Additionally, AE‐ILD developed mostly in the first or second courses (19/30, 63.3%) during chemotherapy. The median time from initiating last line chemotherapy to AE‐ILD was 41 days. Regarding therapeutic regimens, carboplatin‐paclitaxel containing regimens (10 patients) and docetaxel (7 patients) were mostly used. At the time of AE‐ILD development, all patients were evaluated using chest and abdominal CT. Pertaining to the intrathoracic tumor response from baseline or best response according to RECIST version 1.1 at AE‐ILD diagnosis, most patients (18 patients, 60.0%) presented with progressive disease while 11 patients (36.7%) experienced stable disease. Only one patient experienced a partial response. With reference to the tumor response of thoracic lesions from baseline to AE‐ILD diagnosis, the waterfall plot showed that almost all patients (27/30, 90.0%) did not experience any tumor shrinkage, as shown in Figure 2.

**TABLE 2:** Treatment regimen and disease activity at the time of development of AE‐ILD

	All patients (*n* = 30)	NSCLC (*n* = 23)	NEC (*n* = 7)
Treatment line			
First/second/third/fourth or later	8/11/7/4	7/7/6/3	1/4/1/1
Courses of treatment			
First/second/third/fourth/fifth or later	13/6/5/3/3	12/5/2/2/2	1/1/3/1/1
Interval from initiating last line chemotherapy to AE‐ILD development (day)	41 (5–426)	27 (5–168)	64 (20–426)
Therapeutic regimen			
Carboplatin and paclitaxel containing	10	8	2
Carboplatin, paclitaxel	5	4	1
Carboplatin, nab‐paclitaxel	3	2	1
Carboplatin, paclitaxel, bevacizumab	2	2	0
Docetaxel	7	7	0
S‐1	3	3	0
Vinorelbine	3	3	0
Carboplatin, etoposide	2	0	2
Pemetrexed	2	2	0
Irinotecan	1	0	1
Amrubicin	1	0	1
Nogitecan	1	0	1
Tumor response according to RECIST			
PR/SD/PD	1/12/17	0/10/13	1/2/4

Abbreviations: AE‐ILD, acute exacerbation‐interstitial lung disease; NEC, neuroendocrine cell carcinoma; NSCLC, non‐small cell lung cancer; PD, progressive disease; PR, partial response; RECIST, Response Evaluation Criteria in Solid Tumors (version 1.1); SD, stable disease.

### Overall survival

**FIGURE 3:**
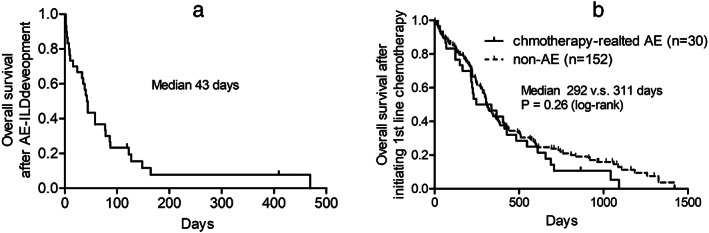
Overall survival after AE‐ILD development (a) and (b) after initiating first‐line chemotherapy. AE‐ILD, acute exacerbation‐interstitial lung disease.

Among 30 patients who developed chemotherapy‐related AE‐ILD, 10 patients died within 4 weeks of AE‐ILD development, whereas 20 patients survived over 4 weeks of AE‐ILD development. At the data cutoff, almost all patients (28 patients) died and the prognosis after AE‐ILD development was very poor. The median OS after AE‐ILD development was 43 days as shown in Figure 3a. With regards to OS after initiating 1st line chemotherapy, the median OS were 292 days in all patients who developed chemotherapy‐related AE‐ILD. Of note, the median OS in patients who did not develop AE‐ILD during their clinical courses was 311 days, and the difference was not significant (Figure 3b, *p* = 0.26). The patient characteristics at the time of initiation of first‐line chemotherapy and treatment histories are shown in Table S1.

## DISCUSSION

The present study showed the following two findings. First, at the time of AE‐ILD development, the majority of patients (18 patients, 60.0%) experienced progressive disease and only one patient (3.3%) experienced a partial response. Notably, almost all patients (27 patients, 90.0%) did not exhibit any tumor shrinkage of the thoracic lesions. Second, most of AE‐ILD occurred during second‐line‐ or later‐line cytotoxic chemotherapy (22/30, 73.3%), and within second courses of treatment (19/30, 63.3%). To the best of our knowledge, this is the first study investigating the disease activity of lung cancer at the time of AE‐ILD development.

In the present study, among 30 patients who developed AE‐ILD, majority of the patients (18 patients, 60.0%) presented with progressive disease according to the RECIST criteria, and almost all patients (27 patients, 90.0%) did not exhibit any tumor shrinkage of the thoracic lesions. These results may indicate that uncontrolled lung cancer is correlated with AE‐ILD development. To our knowledge, there have been eight prospective studies which have investigated the safety and efficacy of first‐line cytotoxic chemotherapy for lung cancer patients with ILD, as shown in Table 3.^9^, ^10^, ^11^, ^12^, ^13^, ^25^, ^26^, ^27^ These studies employed varying therapeutic regimens and slightly different definitions of AE‐ILD in terms of the need for decreased partial pressure of oxygen in arterial blood; all studies showed a low rate of progressive disease (3.0%–19.0%) and good disease control rate (66%–98.2%) with acceptable rates of AE‐ILD (range, 1.6%–12.1%). In contrast, there were four retrospective studies in total. These studies showed a high progressive disease rate (33.3%–69.6%)^14^, ^15^, ^16^, ^26^ and low response rate (8.6%–33%).^14^, ^15^, ^16^, ^28^ Of note, all four studies showed a relatively higher rate of development of AE‐ILD (12.0–27%). Although AE‐ILD has been generally discussed in terms of drug‐specific adverse effects,^9^, ^10^, ^11^, ^12^, ^13^, ^14^, ^15^, ^16^, ^25^ these reports and the results of our study indicate that uncontrolled lung cancer is correlated with the development of AE‐ILD.

**TABLE 3:** Comparison of previous studies on cytotoxic chemotherapy for lung cancer patients with ILD

Author		*N*	Treatment line	Regimen	AE‐ILD (%)	PD rate	DCR	ORR	mPFS
All prospective studies									
Minegishi et al.^12^	2011	18	First‐line	Cb, w‐Pac	1 (5.6%)	11.1%	83.3%	61%	5.3
Sekine et al.^11^	2016	21	First‐line	Cb, S‐1	2 (9.5%)	19.0%	66%	33%	4.2
Hanibuchi et al.^25^	2018	33	First‐line	Cb, S‐1	2 (6.1%)	15.2%	78.8%	33.3%	4.8
Fukuizumi et al.^10^	2019	33	First‐line	Cb, w‐Pac	4 (12.1%)	3.0%	94.0%	69.7%	6.3
Kenmotsu et al.^13^	2019	96	First‐line	Cb, nabPac	4 (4.3%)	18.5%	76.1%	51%	6.2
Asahina et al.^9^	2019	36	First‐line	Cb, nabPac	2 (5.6%)	8.3%	88.9%	55.6%	5.3
Otsubo et al.^26^	2022	121	First‐line	Cb, nabPac, Nin	5 (4.1%)	1.8%	98.2%	69%	6.2
		122	First‐line	Cb, nabPac	2 (1.6%)	11.2%	88.8%	56%	5.5
Sakashita et al.^27^	2022	25	First‐line	Cb, nabPac	1 (4.0%)	4.0%	88%	44%	5.8
All retrospective studies									
Shukuya et al.^14^	2010	15	First‐line	Cb, w‐Pac	4 (27%)	33.3%	53%	33%	2.5
Kato et al.^28^	2014	25	Second‐line	Pemetrexed	3 (12%)	N.D.	72.3%	12.0%	2.9
Enomoto et al.^16^	2015	23	Second or later	Topotecan	5 (21.7%)	69.6%	30.4%^a^	21.7%^a^	N.E.
Watanabe et al.^15^	2015	35	Second‐line	DOC	4 (14.3%)	60.0%	37.1%	8.6%	1.6

Abbreviations: AE, acute exacerbation; DCR, disease control rate; ILD, interstitial lung disease; N.E., not evaluated; ORR, overall response rate; OS, overall survival; PD, progressive disease; PFS, progression‐free survival.

^a^ Personal communication; Nin, nintedanib.

It remains unclear why lung cancer was uncontrolled in most of our patients at AE‐ILD development. However, uncontrolled lung cancer may have the following two adverse effect on ILD. First, immune activations during disease progression of lung cancer may trigger AE‐ILD. Generally, patients with advanced lung cancer are reported to be in a state of immune activation.^29^, ^30^, ^31^ Further, lung cancer patients with ILD and high level of serum C‐reactive protein before chemotherapy were more likely to develop AE‐ILD.^32^, ^33^ In fact, our study showed that serum CRP level was relatively high at initiating last course of chemotherapy. Second, decreased FVC accompanied by uncontrolled lung cancer may also be a risk factor for AE‐ILD. Uncontrolled lung cancer potentially causes bronchial obstruction, the emergence of pleural effusion, and a reduction in performance status, which lead to decreased FVC. A retrospective study showed that low FVC was a risk factor for AE‐ILD in lung cancer patients with ILD.^17^

Although this study contains some important findings, there were three limitations. First, this was a small‐sized retrospective study at a single institution, which potentially leads to selection bias. The current study employed multiple chemotherapeutic agents of various treatment lines which showed diverse response rates and the incidence rate of AE‐ILD. These factors make it difficult to perform statistical analysis. Therefore, future large‐scale studies with matching conditions should be performed to confirm our results. Second, other diseases mimicking ILD or AE‐ILD, such as carcinomatous lymphangitis, pulmonary infection or cardiac failure, could not be completely excluded because of the retrospective nature of this study. However, it would be difficult to perform invasive examination due to deteriorated oxygenation. Finally, pseudoprogression could not be completely ruled out, although the present study included AE‐ILD during cytotoxic chemotherapy, not immune‐checkpoint inhibitors.

In conclusion, lung cancer was mostly uncontrolled with cytotoxic chemotherapy at the time of AE‐ILD development. Although AE‐ILD during chemotherapy has been generally discussed in terms of drug‐specific adverse effects, uncontrolled lung cancer may be also correlated with AE‐ILD development.

## CONFLICT OF INTEREST

All authors declare no conflict of interest.

## Supporting information


**Table S1**. Patient characteristics at the time of initiating 1st line chemotherapy and subsequent treatment histories.
