# Supplementary material for: Disease activity of lung cancer at the time of acute exacerbation of interstitial lung disease during cytotoxic chemotherapy
Source: Thorac Cancer. 2022 Jul 15;13(17):2443–9. doi: 10.1111/1759-7714.14566 (PMC9436679; doi:10.1111/1759-7714.14566)
Supplement: Supplementary file 1 — Table S1. Patient characteristics at the time of initiating 1st line chemotherapy and subsequent treatment histories. [file TCA-13-2443-s001.docx]

| Supplementary Table Patient characteristics at the time of initiating 1^st^ line chemotherapy and subsequent treatment histories | | | |
| --- | --- | --- | --- |
|  | AE-ILD  (n = 30) | Non-AE-ILD  (n = 152) | P value |
| Sex (Male/ female) | 30/ 0 | 134/ 18 | 0.047 |
| Age (range [years]) | 71 (55–79) | 72 (44–87) | 1.00 |
| PS (0-1/ >2) | 28/ 2 | 126/ 26 | 0.17 |
| Smoking history (Current or former/ Never)  Pack-year | 30/ 0  45.5 (20–100) | 151/ 1  45 (0–216) | 1.00  0.76 |
| Radiological classification (UIP/ non-UIP) | 20/ 10 | 80/ 72 | 0.17 |
| Cause of ILD  Idiopathic / CTD/ Pneumoconiosis | 29/ 1/ 0 | 147/ 4/ 1 | 0.89* |
| Pathology (Ad/ Sq/ NOS/ NEC) | 10/ 7 /6/ 7 | 56/ 47/ 8/ 41 | 0.051* |
| Stage (Ⅰ/ Ⅱ/ Ⅲ/ Ⅳ/ recurrence*) | 0/ 0/ 14/ 13/ 3 | 3 / 9 / 52 /60 /28 | 0.33* |
| Laboratory test  KL-6 (n = 189)  SP-D (n = 117)  CRP (n = 206) | 756 (256–6066)  153.5 (39–524)  1.54 (0.07–10.5) | 673 (234–8170)  124 (18–2474)  1.22 (0.02–24.8) | 0.50  0.28  0.43 |
| Respiratory function test  %FVC (n = 81)  FEV1.0% (n = 81)  %DLCO (n = 67) | 81.9 (55.9–109.1)  76.6 (68–92.9)  74.3 (34.5–93.1) | 87.5 (42.8–138.9)  75.6 (34.9–98.9)  64.4 (17.6–137.7) | 0.46  0.53  0.96 |
| Subsequent treatment history  ICI treatment (Yes/ No)  Thoracic radiotherapy (Yes/ No) | 3/ 27  1/ 29 | 22 / 130  15/ 137 | 0.77  0.48 |

AE-ILD, acute exacerbation-interstitial lung disease; *, postoperative; PS, performance status; UIP, usual interstitial pneumonia; NOS, not-other specified; NEC, neuroendocrine cell carcinoma; CTD, connective tissue disease; KL-6, Krebs von den Lungen-6; SP-D, Surfactant Protein-D; CRP, C reactive protein; ICI, immunocheck inhibitor; *, Chi-squire test
